# Mesenchymal stem cells recruited by castration-induced inflammation activation accelerate prostate cancer hormone resistance via chemokine ligand 5 secretion

**DOI:** 10.1186/s13287-018-0989-8

**Published:** 2018-09-26

**Authors:** Yang Yu, Qingyun Zhang, Chengzhong Ma, Xue Yang, Rui Lin, Hongxiang Zhang, Yan Liu, Zhipeng Han, Jiwen Cheng

**Affiliations:** 1 Department of Urology, Shanghai Tenth People’s Hospital, Tongji University School of Medicine, Shanghai, 200072 China; 2 Department of Urology, Affiliated Tumor Hospital of Guangxi Medical University, Nanning, 530021 China; 3 Department of Urology, Guangxi International Zhuang Medicine Hospital, Nanning, 530021 China; 4 Tumor Immunology and Gene Therapy Center, Eastern Hepatobiliary Surgery Hospital, the Second Military Medical University, Shanghai, 200438 China; 5 The Fifth Department of Chemotherapy, Affiliated Tumor Hospital of Guangxi Medical University, Nanning, 530021 China; 6 Department of Urology, The First Affiliated Hospital of Guangxi Medical University, 6 Shuangyong Road, Nanning, 530021 China

**Keywords:** Mesenchymal stem cell, Cancer stem cell, Tumor microenvironment, Oxidative stress, Castration resistance

## Abstract

**Background:**

Androgen deprivation (AD) as the first-line treatment for advanced prostate cancer (PCa) is insufficient for a long-term effect. Castration resistance remains the greatest obstacle in PCa clinical therapy. Mesenchymal stem cells (MSCs) can migrate into PCa tissues contributing to tumor progression, therefore, in this study we explored the effect of AD on MSC migration to PCa and elicited its importance for the emergence of castration resistance.

**Methods:**

MSC migration assay was performed in several PCa cells (LNCaP, VCaP, and 22Rv1) using in-vivo and in-vitro approaches. Reactive oxygen species generation was evaluated by fluorescence assay. IL-1β was analyzed by immunohistochemistry, and neutralization experiments were conducted using neutralization antibody. Stem markers (CD133, CD44, and SOX2) were quantified by real-time PCR analysis. The concentration of chemokine ligand 5 was measured by enzyme-linked immunosorbent assay and small hairpin RNA was used for functional analyses.

**Results:**

AD could significantly contribute to PCa recruitment of MSCs in vivo and in vitro. AD-induced oxidative stress could promote the inflammatory response mediated by IL-1β secretion via activating the NF-κB signaling pathway. Moreover, *N*-acetylcysteine could significantly inhibit MSC recruitment to PCa sites when AD is performed. Furthermore, we found MSCs could increase stemness of PCa cells via promoting chemokine ligand 5 secretion in the AD condition, and consequently accelerate emergence of castration resistance.

**Conclusions:**

Our results suggest that castration in clinical PCa therapy may elicit oxidative stress in tumor sites, resulting in increased MSC migration and in tumor cell growth in an androgen-independent manner. Blocking MSC migration to the tumor may provide a new potential target to suppress castration-resistant PCa emergence.

**Electronic supplementary material:**

The online version of this article (10.1186/s13287-018-0989-8) contains supplementary material, which is available to authorized users.

## Background

Prostate cancer (PCa) is the most commonly diagnosed cancer in men, and the second leading cause of male cancer-associated mortality in the United States [1]. In China, PCa shows an increasing incidence and mortality in recent years [2]. PCa progresses from prostatic intraepithelial neoplasia through locally invasive adenocarcinoma to hormone-resistant metastatic carcinoma. Early staged and localized PCa can be well controlled by prostatectomy or radiotherapy. For locally advanced and metastatic PCa, androgen deprivation therapy (ADT) is typically employed as the first-line treatment [3]. However, most initially androgen-sensitive PCa would develop into castration-resistant prostate cancer (CRPC) after ADT within 12–18 months [4]. So, the transition of androgen-dependent PCa cells to androgen-independent growth during ADT is the main obstacle for advanced PCa treatment.

Recently, considerable efforts toward elucidating the mechanisms of CRPC occurrence suggested that the tumor microenvironment (TME) may play a key role in response to ADT [3, 5–7]. The TME is constituted of tumor epithelial cells and stroma including mesenchymal stem cells (MSCs), also called multipotent mesenchymal stromal cells, inflammatory cells, and fibroblasts [8, 9]. Hypoxia in the TME is a cyclical event and perpetuates the inflammatory response by ensuring a constant production of angiogenic and inflammatory mediators [10]. Hypoxic conditions result in reactive oxygen species (ROS) generation and oxidative stress, which can increase DNA damage in neighboring cells and lead to tissue damage [11]. ROS also increase production of inflammatory mediators inducing inflammatory response in the TME [12]. MSCs, as a vital component of the TME, are a subset of nonhematopoietic stem cells existing in the bone marrow [13]. MSCs can be recruited from the bone marrow to areas of injured tissue and inflammation and then induce peripheral tolerance, promoting damaged cell survival [14]. MSCs also have an innate tropism for tumor tissue in response to the inflammatory microenvironment present in malignant lesions [15] and contribute to various tumor progressions, including PCa [9, 16, 17]. Currently, many studies have shown that ADT increases tumor cell hypoxia in PCa [18, 19]. However, the alteration of MSC migration to PCa tissues and the inflammatory response induced by castration in the TME remain poorly understood.

In this study, we first investigated the contribution of androgen deprivation (AD) in MSC recruitment to PCa using in-vivo and in-vitro approaches, and revealed that castration-induced inflammation activation in response to oxidative stress could increase MSC recruitment to tumor sites. We also found that castration-induced cytokine secretion in MSCs increased the prostate cancer stem cell (PCSC) population, accelerating PCa growth transition from androgen dependent to castration resistant. Our results might represent new potential targets in the battle against advanced PCa.

## Methods

### Cell lines and culture

Human prostatic carcinoma cell lines, including LNCaP, VCaP, and 22Rv1 cells, and bone marrow-derived MSCs were purchased from the Cell Bank of Type Culture Collection of Chinese Academy of Sciences, Shanghai Institute of Cell Biology, Shanghai, China. LNCaP and 22Rv1 cells were cultured in RPMI-1640 medium supplemented with 10% fetal bovine serum (FBS), while VCaP cells were grown in Dulbecco’s modified Eagle’s medium (DMEM) supplemented with 10% FBS. MSCs were cultured in MSC basal medium (all from Invitrogen, Carlsbad, CA, USA). MSCs were transfected with the adenoviral vector GFP-mock (Invitrogen). After transfection for about 48 h, MSCs-GFP were collected for further experiments. All cells were cultured at 37 °C in a 5% CO_2_ humidified atmosphere. AD was performed using charcoal-stripped medium for cell culture as described previously [20].

### In-vivo xenograft experiment

Nude mice, 6–8 weeks old, were obtained from the Shanghai Experimental Animal Center of the Chinese Academy of Sciences, Shanghai, China, and housed in pathogen-free conditions. All aspects of the animal care and experimental procedures were in accordance with the Guide for the Care and Use of Laboratory Animals and approved by the Chinese Academy of Sciences’ Committee on Animals. PCa cells were prepared as single-cell suspensions (1 × 10^6^ cells in 200 μl PBS) and subcutaneously administered in the armpit area of nude mice. When tumors grew to approximately 200 mm^3^ in size, the nude mice were castrated via scrotal incision under methoxyflurane anesthesia. The mice (*n* = 6 per group) were then injected with the green fluorescent protein (GFP)-labeled MSCs (MSCs-GFP) through the tail vein every 3 days. Mice were examined every day and tumor growth was evaluated by measuring the length and width of the tumor mass. All tumor-bearing mice survived until they were sacrificed at the end of the experiment; tumors were then removed and dissected quickly for frozen section preparation, or were stored at − 80 °C.

### Oxidative stress parameter estimation

The frozen tumor samples were thawed and homogenized on ice. PCa cells (3 × 10^3^ cells) were plated in 96-well plates and incubated with charcoal-stripped medium with or without 10 nM of dihydrotestosterone for 48 h. Then, 5 mM of CM-H_2_DCFDA (Invitrogen) was added for 30 min in the dark. Intracellular ROS levels were measured as the mean fluorescence intensity (arbitrary units) according to the manufacturer’s instructions. The 4-HNE adduct was estimated in tumor homogenates using an OxiSelect™ HNE ELISA Kit (Cell Biolabs, San Diego, CA, USA) according to the manufacturer’s instructions.

### Transwell assay

The chemotactic effect of PCa cells on MSCs was assayed utilizing 24-well (8-mm pore size) Transwell plates (Cell Biolabs), while six-well (0.4-mm pore size) Transwell plates were used for coculture. The plate was incubated at 37 °C in a humidified atmosphere containing 5% CO_2_ for 48–72 h. For the migration assay, cells invaded through pores to the lower surface were stained with crystal violet dye and the positively stained cells were counted in six random fields under a microscope.

### Real-time quantitative PCR

To quantify mRNA expression of CCL5 and stem cell markers (CD133, CD44, and SOX2) [21], total RNA was isolated using Trizol reagent (Invitrogen) and cDNA synthesis was performed using the Prime Script RT reagent Kit (Takara, Kyoto, Japan) according to the manufacturer’s specifications. Quantitative PCR was performed using the SYBR Green PCR Kit (Applied Biosystems, Carlsbad, CA, USA) according to the manufacturer’s instructions. β-actin was used as an internal control for RNA integrity and loading normalization.

### Western blot analysis

Tissues were lysed in RIPA lysis buffer (Beyotime Institute of Biotechnology, Haimen, China) with 1 mM PMSF. An equal amount of proteins was separated by SDS-PAGE and transferred to nitrocellulose membrane. The membranes were then washed, blocked, and incubated with specific primary antibodies against IκBα, p-IκBα (both from Cell Signaling Technology, USA), CD133, GFP, and β-actin (all from Abcam, Cambridge, MA, USA), followed by incubation with horseradish peroxidase-conjugated secondary antibodies (HuaAn Biotech, Hangzhou, China). Signals were visualized by chemiluminescent detection (Beyotime).

### Histopathology assessment

LNCaP xenografted tumors were sectioned (4 μm thick) and mounted on glass slides, then stained with Meyer’s hematoxylin and eosin (H&E), and immunostained with primary antibodies for IL-1β (Invitrogen). After glass slides were mounted, each sample was observed at a 200× magnification of the microscopic field. For human PCa immunohistochemistry evaluation, tissue sections were immunostained with primary antibodies for SSEA-4 (eBioscience, San Diego, CA, USA), 4-HNE (Abcam), and IL-1β (Invitrogen). Each sample was observed at a 400× magnification of the microscopic field in 10 randomly selected areas. The intensity and extent of staining were evaluated by score, assigning from 0 to 3 where 0 = none, 1 = weak, 2 = intermediate, and 3 = strong. Final scores were computed using a composite of intensity scores multiplied by the extent of staining score. A score of 1–4 was assessed as low expression and 6–9 as high expression.

### Statistical analysis

All of the experiments were repeated at least three times. Final data were expressed as mean ± standard deviation (SD). Student’s *t* test was performed to compare between mean values of two groups using GraphPad Prism 5. Clinically, statistical analysis was performed using SPSS 22.0. Differences between categorical variables were assessed by the chi-square test or Fisher’s exact test. A value of at least *p* < 0.05 was considered statistically significant.

## Results

### Castration increases MSC recruitment and oxidative stress in PCa

**Fig. 1:**
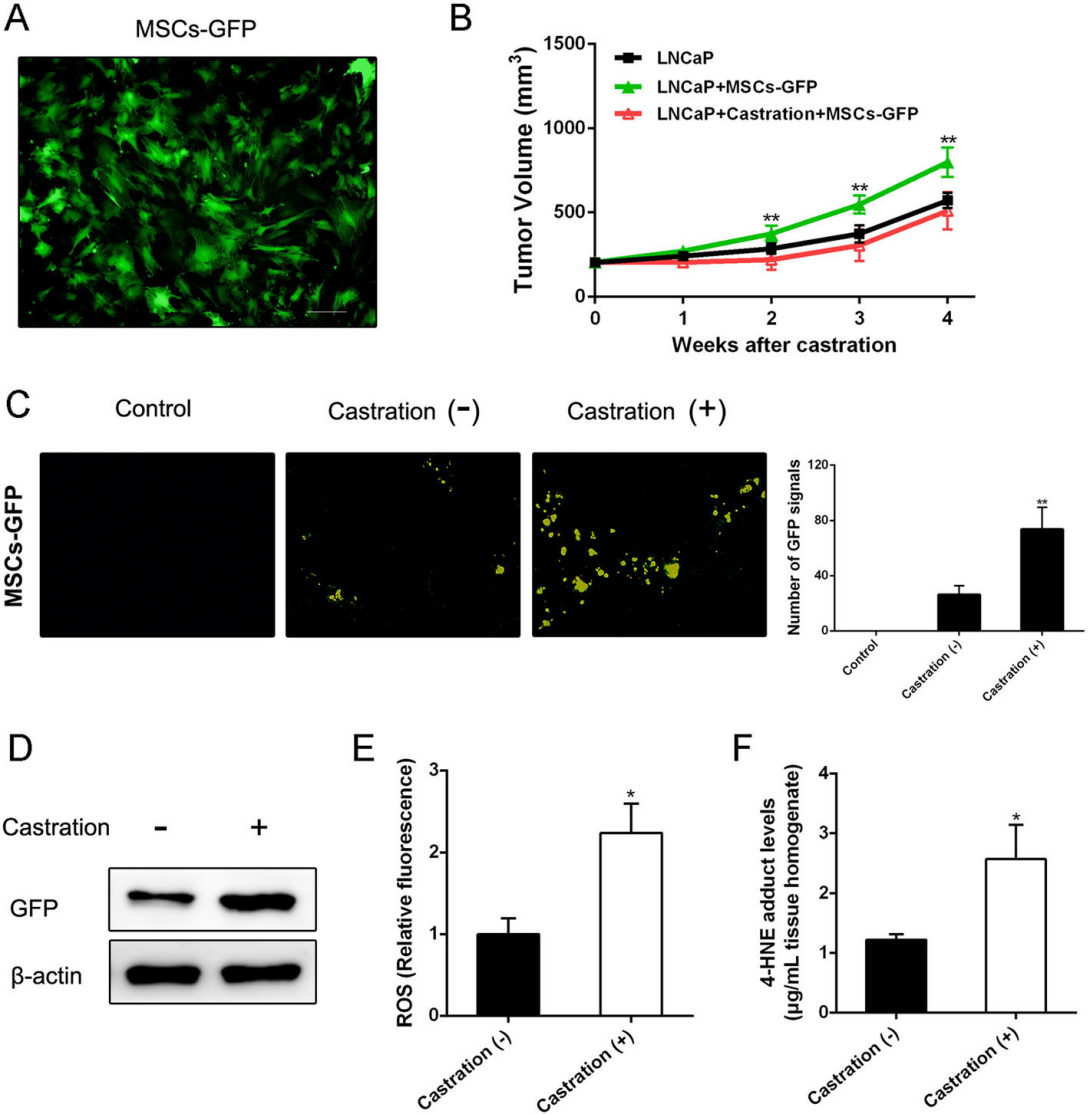
Castration increases PCa recruitment of MSCs and oxidative stress in vivo*.* **a** MSCs transfected with adenoviral vector GFP-mock (Invitrogen). After transfection for about 48 h, MSCs-GFP detected by fluorescence microscope (original magnification: ×200). **b** LNCaP xenografted tumors measured by calipers, then volume calculated using the formula: volume = width^2^ × length × 0.5236. **c** Two weeks after MSCs-GFP injection, tumor tissues removed from mice with castration or not (tumors from untreated mice as control) were embedded in Tissue-Tek OCT compound and snap frozen in liquid nitrogen. Cryostat sections (6 mm thick) prepared using Leica CM1950 cryostat. GFP fluorescence signal analyzed with fluorescence microscope (original magnification: ×200). **d** Western blot analysis of GFP expression in tumor tissues. **e, f** ROS and 4-HNE adduct levels estimated in tumor homogenates to reflect oxidative stress level. *p < 0.05, **p < 0.01. GFP green fluorescent protein, MSC mesenchymal stem cell, ROS reactive oxygen species

To investigate whether castration could affect MSC recruitment, we first infected MSCs with an adenovirus vector to obtain GFP-labeled MSCs (Fig. 1a). Studies were then performed in the LNCaP xenograft mouse model. As shown in Fig. 1b, MSCs could effectively accelerate prostate tumor growth. Significantly, higher numbers of GFP signals in frozen sections were detected in tumors removed from mice suffering castration compared with those without castration (Fig. 1c). Western blot analysis confirmed that GFP protein levels in tumors were substantially increased after mice suffered castration (Fig. 1d). We further tested ROS and 4-HNE adduct levels in tumor tissue samples to confirm whether castration could give rise to oxidative stress. As shown in Fig. 1e, castration induced a clear increase of ROS generation in tumor tissues. Correspondingly, 4-HNE adduct levels in tumor tissues of castrated mice were significantly increased (Fig. 1f).

**Fig. 2:**
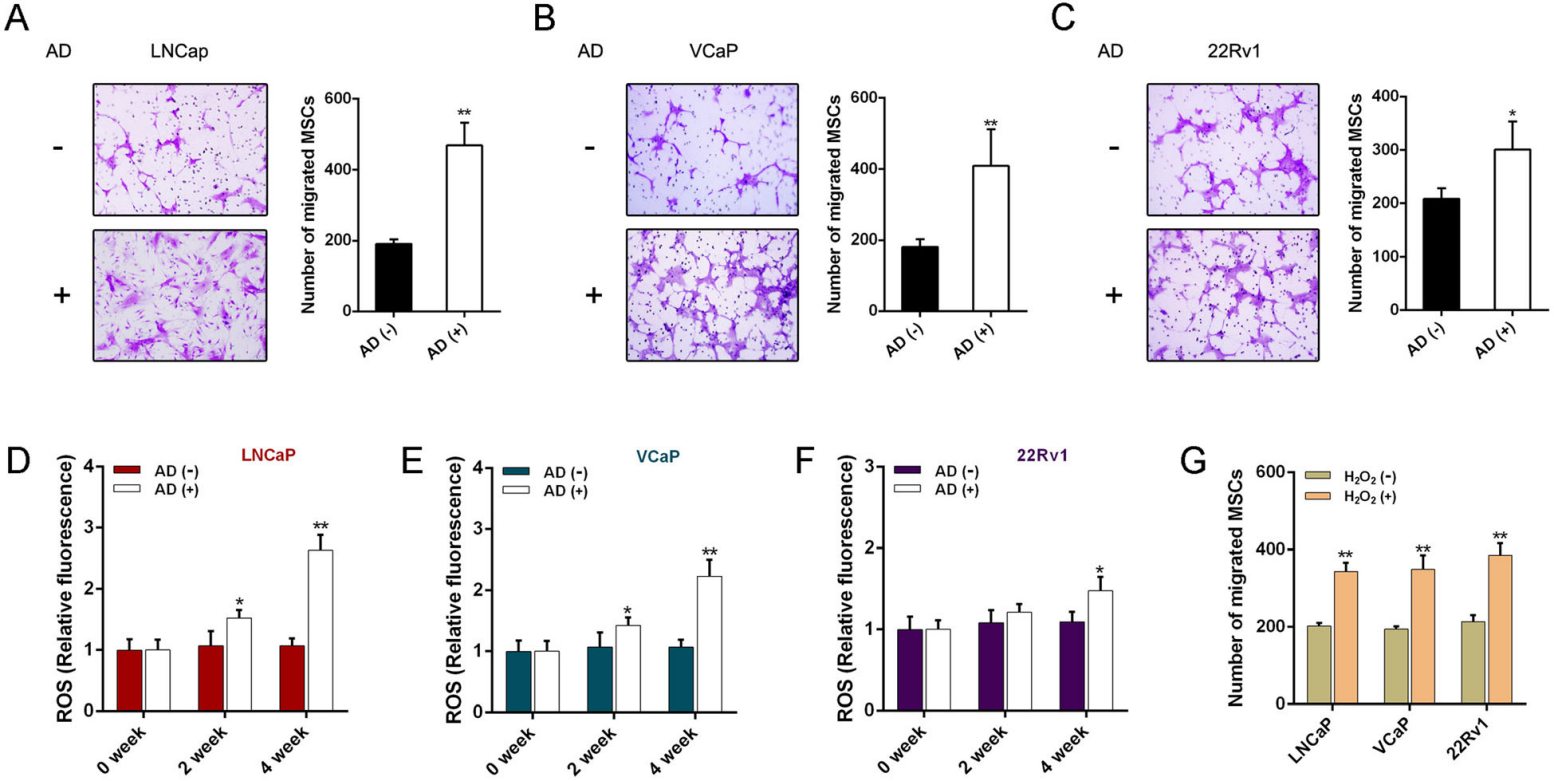
AD increases MSC migration and intracellular ROS level of PCa cells. **a**–**c** Chemotactic effect on MSCs detected in LNCaP, VCaP, and 22Rv1 cells via Transwell assay when AD performed. MSCs (1 × 10^5^ cells) added to upper chamber containing 200 μl of serum-free medium, while PCa cells placed in lower chamber containing 300 μl charcoal-stripped medium with or without 10 nM of dihydrotestosterone (DHT). After 48 h of incubation, representative staining of migrated MSCs presented (left) and quantified (right). **d**–**f** LNCaP, VCaP, and 22Rv1 cells assayed for intracellular ROS levels when AD administrated respectively for 2 and 4 weeks. **g** MSC migration assay results after LNCaP, VCaP, and 22Rv1 cells treated with 10 mM of H_2_O_2_ for 2 h. *p < 0.05, **p < 0.01. AD androgen deprivation, H_2_O_2_ hydrogen peroxide, MSC mesenchymal stem cell, ROS reactive oxygen species

In addition, we also performed in-vitro experiments for migration assays using three human PCa cell lines (LNCaP, VCaP, 22Rv1). As shown in Fig. 2a–c, MSC recruitment was significantly increased when PCa cells suffered AD. Intracellular ROS levels of PCa cells were gradually increased in PCa cells that underwent AD (Fig. 2d–f). We also found that hydrogen peroxide (H_2_O_2_)-treated PCa cells recruited more MSCs, indicating that intracellular ROS increase could directly encourage MSC recruitment (Fig. 2g). These results imply that castration could significantly induce oxidative stress in PCa and increase MSC recruitment.

### AD activates inflammatory response mediated by NF-κB signaling

**Fig. 3:**
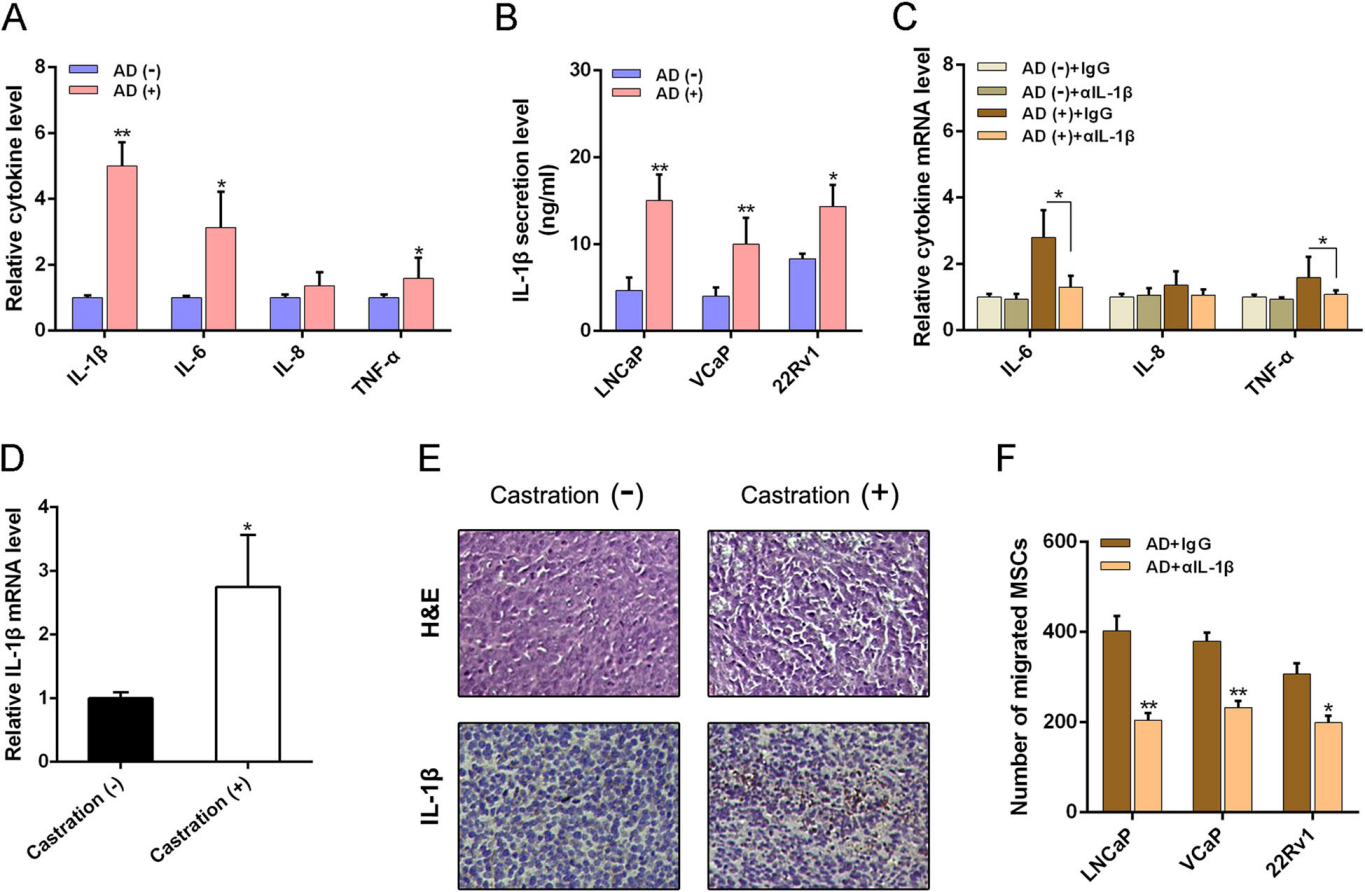
IL-1β is key mediator of AD-induced MSC migration. **a** Cytokine analysis of CM isolated from LNCaP cells suffering AD or not for 48 h, and expression determined by Bio-Plex Pro™ cytokine assay kit (Bio-Rad Laboratories, USA). **b** ELISA performed to examine concentration of IL-1β in CM isolated from LNCaP cells 48 h after AD administration using commercial ELISA kits (R&D Systems, Minneapolis, MN, USA) according to manufacturer’s instructions. **c** RT-PCR analysis of cytokine expression levels in LNCaP cells with or without AD administration, in absence and presence of 1 mg/ml IL-1β neutralization antibody (αIL-1β). **d** RT-PCR analysis of IL-1β expression level in tumor tissues with or without castration. **e** H&E staining and IHC analysis of cross-sections through tumors obtained from mice with or without castration (original magnification: ×200). **f** MSC migration assay results after PCa cells administered with AD, in absence and presence of 1 mg/ml IL-1β neutralization antibody. *p < 0.05, **p < 0.01. AD androgen deprivation, H&E hematoxylin and eosin, IgG immunoglobulin G, IL interleukin, MSC mesenchymal stem cell, TNF tumor necrosis factor

Previous studies have shown evidence that the oxidative stress and inflammation can crosstalk and contribute to each other [12, 22]. Cytokines are major mediators of communication between cells in the inflammatory tumor microenvironment. We then performed a cytokine assay to globally identify inflammatory mediators in the conditioned medium (CM) obtained from LNCaP cells. The most abundant cytokines were IL-1β, IL-6, IL-8, and TNF-α (Fig. 3a) after AD administration. Interestingly, we found consistent upregulation of IL-1β and IL-6 expression in 22Rv1 cells suffering AD (data not shown). We focused on IL-1β since a previous study has identified IL-1β as a new biomarker to evaluate the probability of PCa biochemical recurrence [23]. Next, we confirmed that AD led to the greatest increase secretion of IL-1β in PCa (LNCap, VCaP, and 22Rv1) cells (Fig. 3b). We also found significant downregulation of mRNA expression of IL-6, IL-8, and TNF-α after neutralization of IL-1β by a specific antibody (Fig. 3c), suggesting that IL-1β induction could possibly be an early and vital event during the crosstalk. Consistently, we detected a high IL-1β expression level in PCa tumor tissues obtained from mice suffering castration (Fig. 3d, e). Histological analysis also demonstrated a dramatic change in response to inflammation including low cell density and multinucleated cells with condensed chromatin staining and pyknosis (Fig. 3e). As shown in Fig. 3f, inhibiting IL-1β activity led to a significant suppression in AD-mediated MSC migration increase to PCa cells. Our results suggest that IL-1β plays a crucial role in MSC recruitment in response to AD-induced oxidative stress in PCa.

**Fig. 4:**
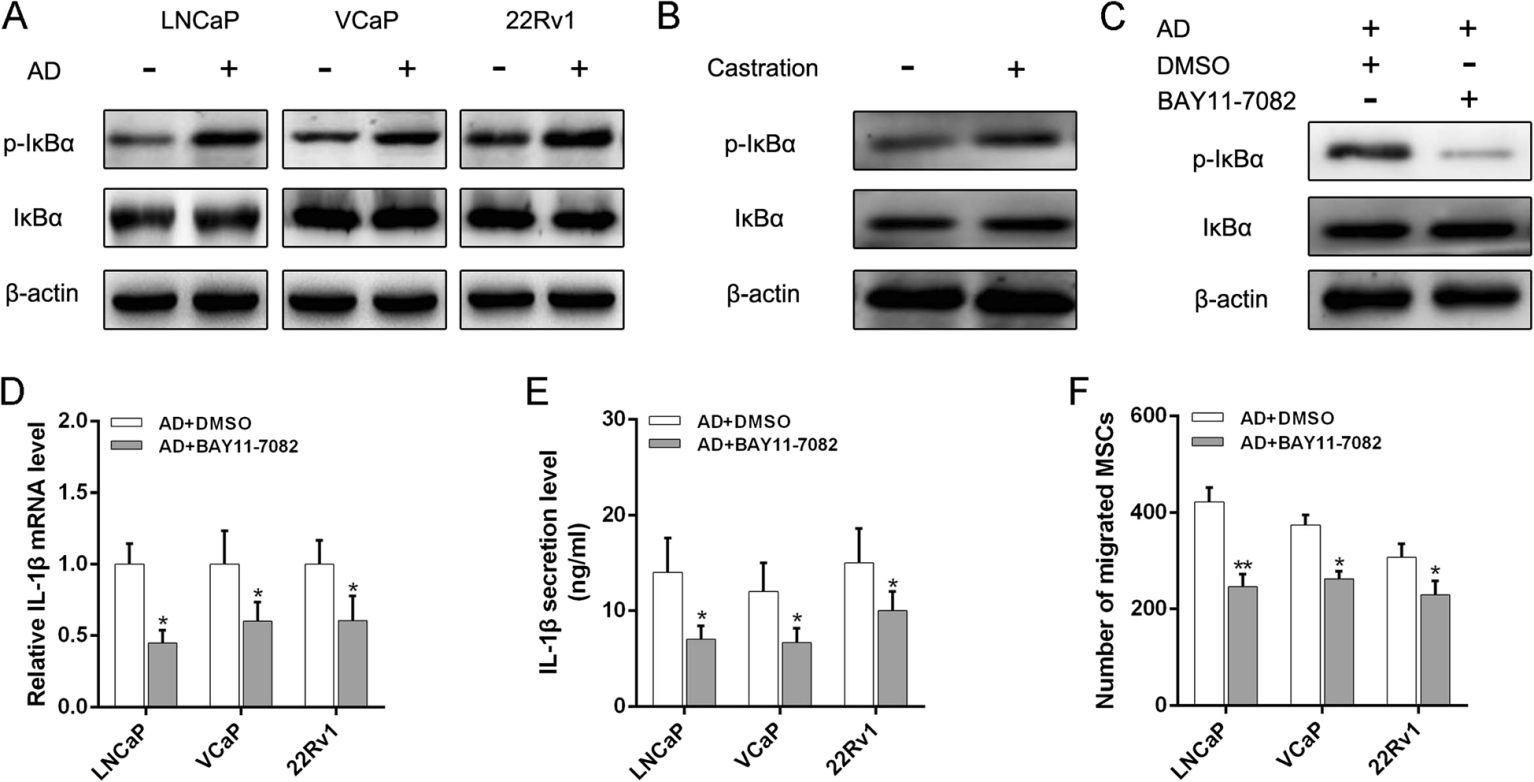
AD increases IL-1β secretion by activating NF-κB signaling pathway. **a** Activation of NF-κB in PCa cells after AD administration detected by western blot analysis of p-IκBα and IκBα. **b** Western blot analysis of NF-κB activation in tumor tissues of mice suffering castration. **c** Inhibition of IκBα phosphorylation by BAY11-7082 (10 μM) confirmed using western blot analysis. **d** RT-PCR analysis of IL-1β expression level in LNCaP cells after AD administration, in absence and presence of BAY11-7082. **e** ELISA performed to examine concentration of IL-1β in CM isolated from LNCaP cells after AD administration, in absence and presence of BAY11-7082. **f** MSC migration assays performed after adding BAY11-7082 to lower chamber when PCa cells suffered from AD. *p < 0.05, **p < 0.01. AD androgen deprivation, BAY11-7082 NF-κB inhibitor, DMSO dimethylsulfoxide, IL interleukin, MSC mesenchymal stem cell

Since an earlier study demonstrated that a constitutively active form of NF-κB promotes a series of inflammatory responses [24], we examined the NF-κB signaling pathway and found an increase of p-IκBα in PCa cells after AD (Fig. 4a). Meanwhile, we detected consistent results in PCa tumor tissues (Fig. 4b). To validate the role of the NF-κB signaling pathway in mediating the AD-induced inflammatory response in PCa, we used BAY11-7082, a NF-κB inhibitor, to determine whether suppression of NF-κB activation might inhibit IL-1β secretion (Fig. 4c). Significantly, BAY11-7082 led to a great reduction in mRNA and protein expression in IL-1β responded to AD (Fig. 4d, e). Consistently, suppressing NF-κB activation led to a significant reduction in AD-mediated MSC recruitment to PCa (Fig. 4f). Taken together, our results indicate that NF-κB signaling pathway-mediated IL-1β secretion plays an important role in AD-induced MSC recruitment to PCa.

### Oxidative stress suppression inhibits MSC recruitment via reducing inflammation activation in PCa

**Fig. 5:**
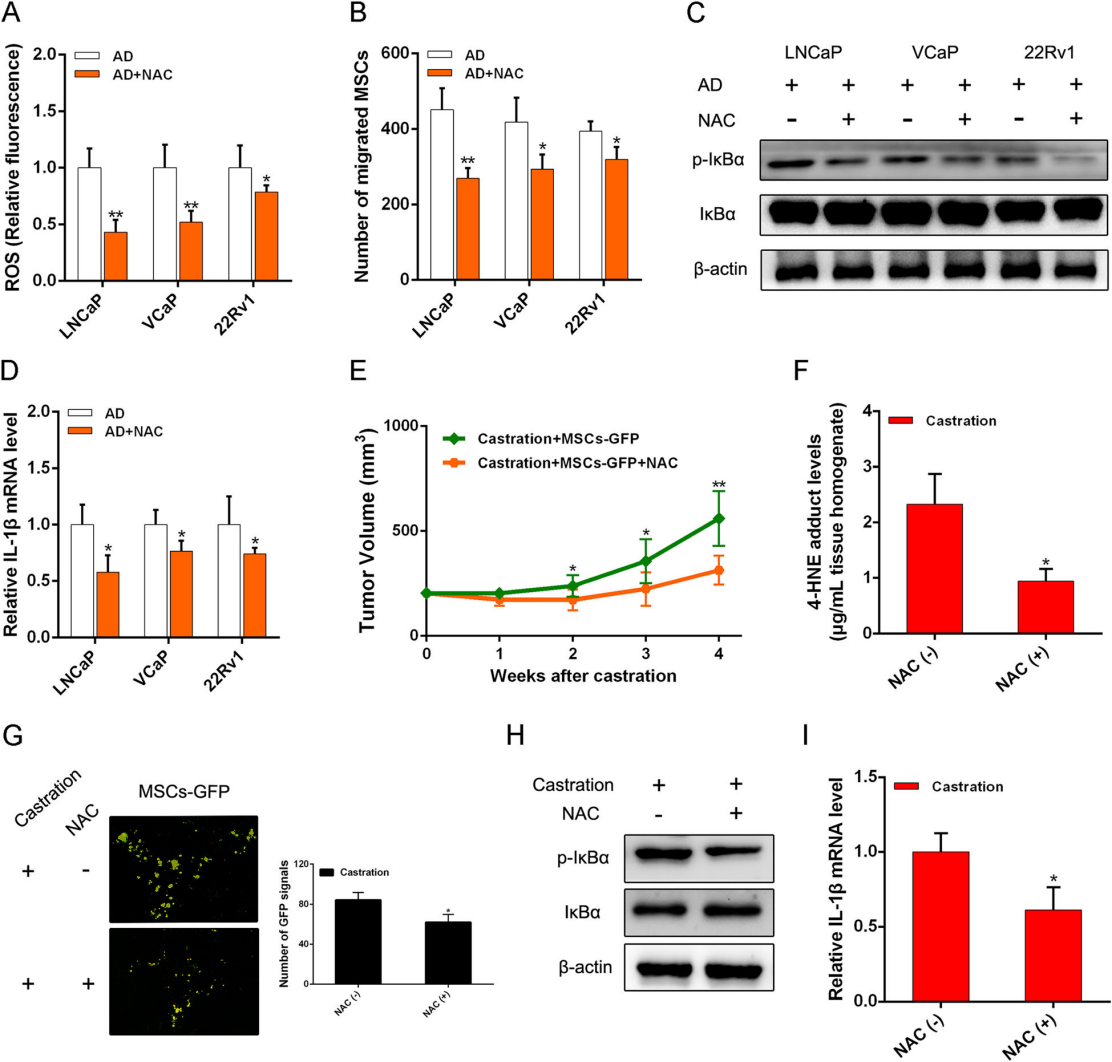
NAC alleviates PCa recruitment of MSCs via oxidative stress suppression. **a** Intracellular ROS levels estimated in PCa cells with AD after 5 mM NAC added to media. **b** PCa cells on MSC migration demonstrated after NAC added in AD. **c** Western blot analysis of NF-κB activation in PCa cells after adding NAC in AD. **d** RT-PCR analysis applied to determine expression of IL-1β in PCa cells after adding NAC. **e** In LNCaP xenograft mouse model, NAC (100 mg/kg) administered intraperitoneally daily after castration. Tumors measured by calipers, then volume calculated. **f** 4-HNE adduct levels determined in tumor homogenates when tumors removed at 4th week. **g** Influence of NAC on migration of MSCs to tumor sites when tumor-bearing mice castrated. **h** Western blot analysis of GFP expression in tumor tissues. **i** Influence of NAC on expression of IL-1β in tumor tissues when mice castrated. *p < 0.05, **p < 0.01. AD androgen deprivation, GFP green fluorescent protein, IL interleukin, NAC *N*-acetylcysteine, ROS reactive oxygen species, MSC mesenchymal stem cell

Next, to confirm that MSC recruitment correlates with oxidative stress caused by AD, PCa cells were exposed to AD and the free-radical scavenger *N*-acetylcysteine (NAC). As shown in Fig. 5a, addition of NAC dramatically abolished the increase of intracellular ROS induced by AD. MSC recruitment increase was also suppressed by NAC in AD (Fig. 5b). Consistently, suppressing ROS led to a significant reduction in NF-κB activation, with a decrease in IL-1β induction (Fig. 5c, d). Meanwhile, results in the LNCaP xenograft mouse model showed that NAC administration led to a significant inhibition of castration-resistant tumor growth and castration-induced oxidative stress (Fig. 5e, f). We speculated that NAC might restrict tumor growth via suppressing MSC migration. The decrease in MSC migration was also detected in PCa tumor tissues when NAC was present, which confirmed our speculation (Fig. 5g). We further detected consistent results that NF-κB activation and IL-1β induction were also suppressed by NAC (Fig. 5h, i). Together, results from in-vitro cell lines and in-vivo mice studies suggest that castration may induce oxidative stress in PCa sites contributing to the increase of MSC migration, which can be abolished by NAC.

### MSCs increase PCa stemness in AD to accelerate CRPC progression

**Fig. 6:**
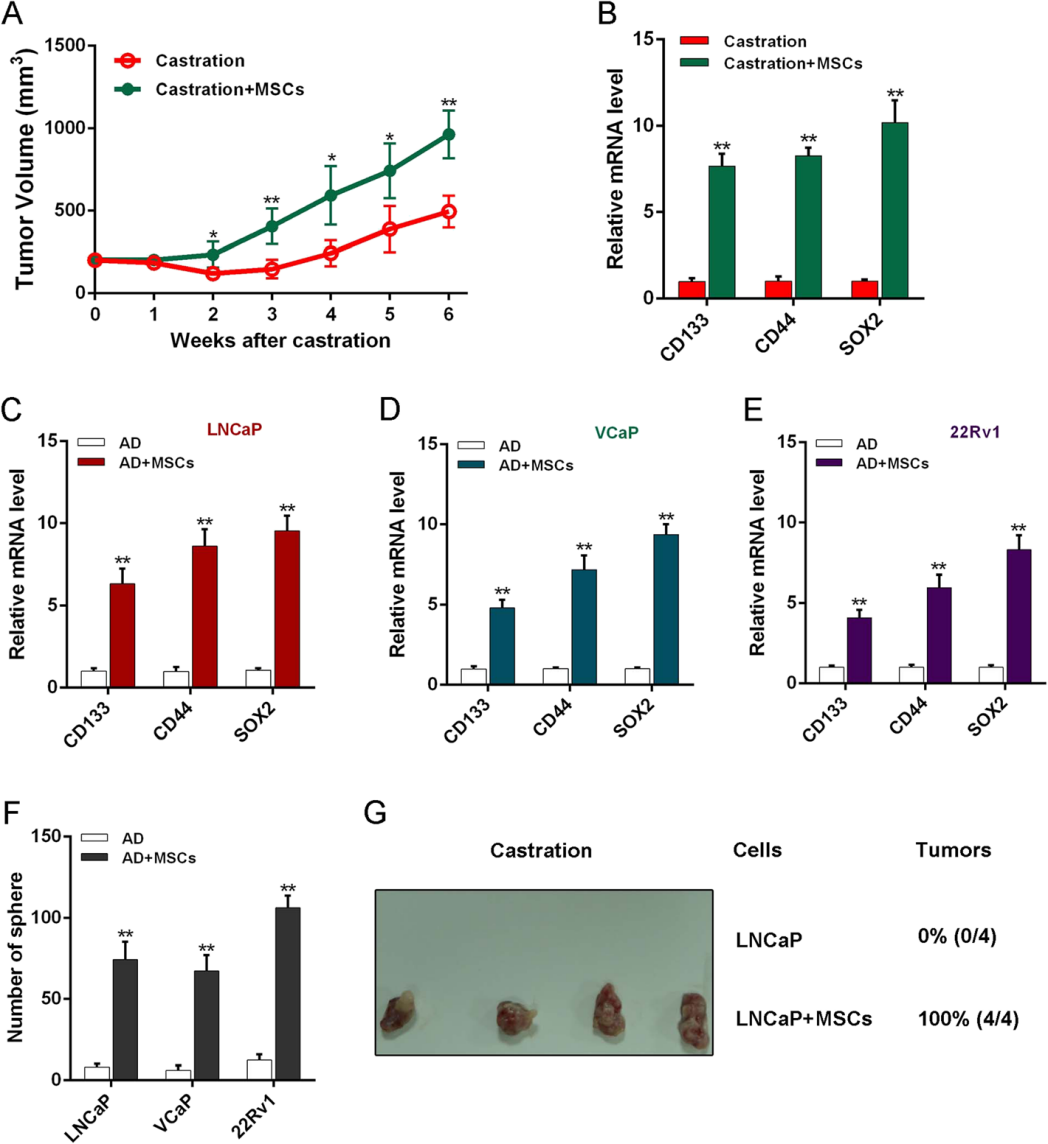
MSCs increase stemness of PCa inducing castration-resistant growth. **a** Castrated mice with LNCaP tumors (*n* = 6 per group) injected with MSCs or not through tail vein every 3 days, tumor volume observed and calculated. After 6 weeks, tumors removed for further experiments. **b** RT-PCR analysis stem marker mRNA expression in tumors. **c**–**e** RT-PCR employed to examine stem marker expression levels of PCa cells. **f** Sphere formation assay of PCa cells after coculture with MSCs for 5 days in AD, then mixed with Matrigel (1:1, v/v), and cultured in 24-well plates for 10 days. **g** PCa cells, after coculture with MSCs for 5 days, subcutaneously injected into armpit area of nude mice and rate of tumor formation observed at day 14. *p < 0.05, **p < 0.01. AD androgen deprivation, MSC mesenchymal stem cell

In addition, we further investigated the effect of MSCs on androgen-deprived PCa growth. Results showed that tumor volumes increased more rapidly in mice with MSC treatment compared with controls by 2 weeks after castration (Fig. 6a). We also found increased expression of stem cell markers in tumor tissues of castrated mice with MSC treatment compared with controls (Fig. 6b and Additional file 1: Figure S1A). As we know, PCSCs as the origin of PCa play a key role in tumor recurrence and treatment resistance [25, 26]. So, we speculated that MSCs might promote CRPC emergence through increasing the PCSC population. Therefore, we next investigated the influence of MSCs on stemness of PCa cells in AD and found that PCa cells upon MSC coculture showed an increased expression of stem cell markers (Fig. 6c–e and Additional file 1: Figure S1B, C). We also performed a sphere formation assay to examine the self-renewal ability of PCa cells, and the results showed that the sphere number was obviously increased when androgen-deprived PCa cells were cocultured with MSCs (Fig. 6f). We then tested the effect of MSCs on tumorigenesis when LNCaP cells were transplanted into mice with castration administration, and after 2 weeks we found that tumors were detected in all mice transplanted with MSCs and LNCaP cells, whereas those transplanted with LNCaP cells only did not form tumors (Fig. 6g). Combining these results, MSCs increased the original PCSC population and self-renewal ability, resulting in a PCa cell proliferation increase in an androgen-independent manner.

### MSCs induce PCa androgen-independent growth depending on chemokine ligand 5 secretion

**Fig. 7:**
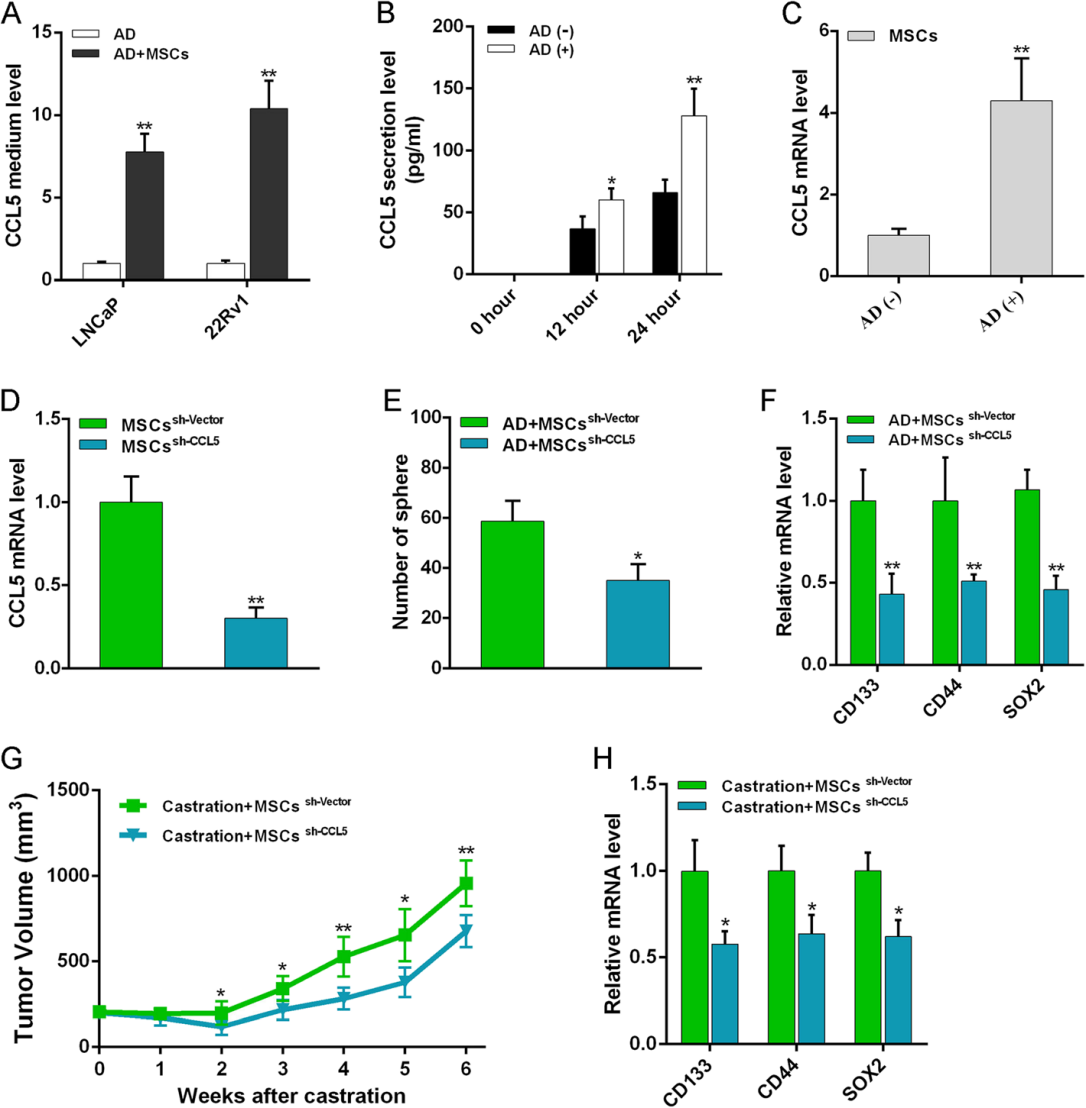
CCL5 essential for PCa hormone resistance induction by MSCs. **a** ELISA kit (R&D Systems, Minneapolis, MN, USA) applied to examine concentration of CCL5 in coculture medium of MSCs and PCa cells in AD. **b** ELISA performed to examine CCL5 secretion of MSCs in AD. **c** RT-PCR analysis of CCL5 expression in MSCs under AD. **d** CCL5 small hairpin RNA (shRNA) oligonucleotides (Neuron Bio, Shanghai, China) with stem-loop structure containing CCL5-target sequence (5′-GTGTGTGCCAACCCAGAGA-3′) used for knockdown analysis. MSCs (1–3 × 10^6^ cells) growing to 50–60% confluence in 10-cm Petri dishes transfected with CCL5-targeting shRNA or their corresponding nontargeting shRNA using lentiviral vectors for 48 h. Effective knockdown effect of CCL5 in MSCs confirmed by RT-PCR measurement. **e** Influence of MSCs on sphere formation assay of PCa cells with CCL5 knockdown in AD. **f** mRNA expression of stem markers in LNCaP cells tested when cocultured with CCL5-knocked MSCs in AD. **g** LNCaP tumor volume observed again to evaluate effect of CCL5 on tumor castration-resistant growth. **h** Expression of stem markers also examined after tumor removed at 6th week after CCL5-knocked MSC treatment. *p < 0.05, **p < 0.01. AD androgen deprivation, CCL-5 chemokine ligand 5, MSC mesenchymal stem cell

It has been reported that MSCs can secrete chemokine ligand 5 (CCL5) to mediate PCSC formation [27], so we tested the CCL5 level in the CM obtained from the culture of androgen-deprived PCa cells with MSCs or not. As shown in Fig. 7a, CCL5 was dramatically increased in androgen-deprived PCa cells cocultured with MSCs. We then investigated the effect of AD on secretion of CCL5 in MSCs, and found that CCL5 secretion increased in a time-dependent manner when AD was performed (Fig. 7b). We also validated significantly increased CCL5 mRNA expression in androgen-deprived MSCs (Fig. 7c). Next, CCL5 was silenced in MSCs by shRNA and RT-PCR analysis showed that sh-CCL5 exhibited the most effective knockdown effect (Fig. 7d). We found that the increased sphere formation ability of androgen-deprived PCa cells upon MSC coculture was obviously suppressed when MSCs suffered CCL5 knockdown (Fig. 7e). Effects of sh-CCL5 on blocking stemness-associated gene expressions were also demonstrated (Fig. 7f). In addition, results in the xenotransplant tumor model showed that CCL5 knockdown significantly inhibited the effect of MSCs on increasing the volume of prostate tumor in AD (Fig. 7g). We also validated the decreased expression of stem cell markers in tumor tissues (Fig. 7h). These results indicate that CCL5 secretion in MSCs is essential for the PCSC population increase in AD, resulting in PCa progression to castration resistance.

## Discussion

Previous studies have proved that, after an initial response, PCa cells can adapt to AD via altering the local TME [3, 5]. Therefore, the effect of AD on tumor inflammatory microenvironment alteration and MSC recruitment in PCa tissues attracted our attention. In the current study, we evaluated the influence of AD on the migration of MSCs to the prostate TME. We found that PCa suffering AD could recruit more MSCs to PCa sites in both in-vivo and in-vitro experiments (Figs. 1c and 2a–c).

The TME has been reported to perform critical roles for cancer progression. The interaction between epithelial cells and stromal cells in the TME ranges from supporting tumor cell proliferation to inducing tumorigenesis and metastasis [28–30]. Evidence has suggested that infiltrating macrophage and cancer cell interaction in the prostate TME can promote castration resistance via a nuclear receptor derepression pathway [7]. Prostate fibroblasts were also demonstrated to facilitate PCa development of castration resistance and metastatic potential [31]. As we know, MSCs have been implicated in tumorigenesis through multiple mechanisms, including promoting proliferation, angiogenesis, and metastasis, in addition to the generation of an immunosuppressive microenvironment assisting tumor escape from immunosurveillance [32–34]. However, the role of MSCs in development of CRPC during ADT remains poorly clarified. It has been reported that MSCs can secrete a large number of cytokines to affect cancer stem cell (CSC) formation and mediate tumor development [35, 36]. CSCs refer to a subset of tumor cells that has the ability to self-renew and differentiate, continually sustaining tumorigenesis, progression, and metastasis [37]. They are also thought to be the main force behind resistance to chemotherapy and radiotherapy [38, 39]. So, we speculated that CSCs induced by MSCs might play a key role in emergence of castration resistance. In the present study, we validated that MSCs increased the population and self-renewal ability of the original PCSCs via a large amount of CCL5 secretion in AD, accelerating the progression to CRPC (Figs. 6 and 7).

Nowadays, although encouraging new drugs like abiraterone and enzalutamide have been developed recently, CRPC is still incurable by current treatment strategies. We think that blocking MSC migration to PCa might be an effective therapy during progression to castration resistance. So, the mechanism for increased MSC migration to PCa when AD is performed must be elucidated to find the successful target. In the present study, in-vivo and in-vitro studies confirmed that AD could significantly induce oxidative stress and inflammation activation mediated by NF-κB signaling, promoting MSC migration to PCa sites (Figs. 3 and 4). Furthermore, NAC administration could effectively alleviate MSC migration to PCa when AD is performed, and, as expected, PCa tumor growth was also inhibited in castrated mice with MSC treatment (Fig. 5).

In summary, our results reveal that castration could effectively contribute to PCa recruitment of MSCs. The data also show that this effect is dependent on inflammation activation mediated by castration-induced oxidative stress via activating the NF-κB signaling pathway. Antioxidant application could significantly inhibit MSC recruitment to PCa sites when AD is performed. Furthermore, we found MSCs could increase the stemness of PCa cells via promoting chemokine ligand 5 secretion in AD, and consequently accelerate emergence of castration resistance.

## Conclusions

Our current study showed that castration could induce PCa oxidative stress and then increase the inflammatory response, contributing to MSCs recruited to PCa sites. In AD, MSCs could promote PCa castration resistance via secreting chemokine ligand 5 and increasing the PCSC population. According to our study, MSCs may be involved in the pathogenesis of CRPC in human PCa, indicating that castration at the same time as targeting oxidative stress using an antioxidant to block PCa recruitment of MSCs would be a new potential treatment strategy to prevent the progression to CRPC.

## Additional file

Additional file 1: **Figure S1.** MSCs increase stem marker expression of PCa. **A.** Western blot analysis of stem marker (CD133) expression in PCa tumor tissues. **B.** Western blot analysis of stem marker (CD133) expression in LNCaP cells. **C.** LNCaP cells stained with primary antibodies anti-CD133 (Abcam). Sections counterstained with DAPI (Beyotime) for nuclei staining. CD133 expression determined by fluorescence microscope. Typical photographs presented (original magnification: ×200) (JPG 205 kb)

## Abbreviations

AD: Androgen deprivation
ADT: Androgen deprivation therapy
CCL5: Chemokine ligand 5
CRPC: Castration-resistant prostate cancer
CSC: Cancer stem cell
DMEM: Dulbecco’s modified Eagle’s medium
ELISA: Enzyme-linked immunosorbent assay
FBS: Fetal bovine serum
GFP: Green fluorescent protein
H_2_O_2_: Hydrogen peroxide
MSC: Mesenchymal stem cell
NAC: *N*-acetylcysteine
PCa: Prostate cancer
PCSC: Prostate cancer stem cell
ROS: Reactive oxygen species
RT-PCR: Real-time quantitative PCR
shRNA: Small hairpin RNA
TME: Tumor microenvironment

## References

[1] Siegel RL, Miller KD, Jemal A (2017). Cancer statistics, 2017. CA Cancer J Clin.

[2] Chen W, Zheng R, Baade PD, Zhang S, Zeng H, Bray F (2016). Cancer statistics in China, 2015. CA Cancer J Clin.

[3] Karantanos T, Evans CP, Tombal B, Thompson TC, Montironi R, Isaacs WB (2015). Understanding the mechanisms of androgen deprivation resistance in prostate cancer at the molecular level. Eur Urol.

[4] Gulley J, Figg WD, Dahut WL (2003). Treatment options for androgen-independent prostate cancer. Clin Adv Hematol Oncol.

[5] Montgomery RB, Mostaghel EA, Vessella R, Hess DL, Kalhorn TF, Higano CS (2008). Maintenance of intratumoral androgens in metastatic prostate cancer: a mechanism for castration-resistant tumor growth. Cancer Res.

[6] Locke JA, Fazli L, Adomat H, Smyl J, Weins K, Lubik AA (2009). A novel communication role for CYP17A1 in the progression of castration-resistant prostate cancer. Prostate.

[7] Zhu P, Baek SH, Bourk EM, Ohgi KA, Garcia-Bassets I, Sanjo H (2006). Macrophage/cancer cell interactions mediate hormone resistance by a nuclear receptor derepression pathway. Cell.

[8] Sun Z, Wang S, Zhao RC (2014). The roles of mesenchymal stem cells in tumor inflammatory microenvironment. J Hematol Oncol.

[9] Brennen WN, Denmeade SR, Isaacs JT (2013). Mesenchymal stem cells as a vector for the inflammatory prostate microenvironment. Endocr Relat Cancer.

[10] Spaeth E, Klopp A, Dembinski J, Andreeff M, Marini F (2008). Inflammation and tumor microenvironments: defining the migratory itinerary of mesenchymal stem cells. Gene Ther.

[11] Laconi E (2007). The evolving concept of tumor microenvironments. BioEssays.

[12] Qu Y, Oyan AM, Liu R, Hua Y, Zhang J, Hovland R (2013). Generation of prostate tumor-initiating cells is associated with elevation of reactive oxygen species and IL-6/STAT3 signaling. Cancer Res.

[13] Deans RJ, Moseley AB (2000). Mesenchymal stem cells: biology and potential clinical uses. Exp Hematol.

[14] Sotiropoulou PA, Papamichail M (2007). Immune properties of mesenchymal stem cells. Methods Mol Biol.

[15] Nakamizo A, Marini F, Amano T, Khan A, Studeny M, Gumin J (2005). Human bone marrow-derived mesenchymal stem cells in the treatment of gliomas. Cancer Res.

[16] Ye H, Cheng J, Tang Y, Liu Z, Xu C, Liu Y (2012). Human bone marrow-derived mesenchymal stem cells produced TGFbeta contributes to progression and metastasis of prostate cancer. Cancer Investig.

[17] Karnoub AE, Dash AB, Vo AP, Sullivan A, Brooks MW, Bell GW (2007). Mesenchymal stem cells within tumour stroma promote breast cancer metastasis. Nature.

[18] Tong D, Liu Q, Liu G, Yuan W, Wang L, Guo Y (2016). The HIF/PHF8/AR axis promotes prostate cancer progression. Oncogene.

[19] Johansson A, Rudolfsson SH, Kilter S, Bergh A (2011). Targeting castration-induced tumour hypoxia enhances the acute effects of castration therapy in a rat prostate cancer model. BJU Int.

[20] Tso CL, McBride WH, Sun J, Patel B, Tsui KH, Paik SH (2000). Androgen deprivation induces selective outgrowth of aggressive hormone-refractory prostate cancer clones expressing distinct cellular and molecular properties not present in parental androgen-dependent cancer cells. Cancer J.

[21] Kasper S (2008). Exploring the origins of the normal prostate and prostate cancer stem cell. Stem Cell Rev.

[22] Zhong P, Wu L, Qian Y, Fang Q, Liang D, Wang J (2015). Blockage of ROS and NF-kappaB-mediated inflammation by a new chalcone L6H9 protects cardiomyocytes from hyperglycemia-induced injuries. Biochim Biophys Acta.

[23] Eiro N, Bermudez-Fernandez S, Fernandez-Garcia B, Atienza S, Beridze N, Escaf S (2014). Analysis of the expression of interleukins, interferon beta, and nuclear factor-kappa B in prostate cancer and their relationship with biochemical recurrence. J Immunother.

[24] Zhong Z, Umemura A, Sanchez-Lopez E, Liang S, Shalapour S, Wong J (2016). NF-kappaB restricts inflammasome activation via elimination of damaged mitochondria. Cell.

[25] Domingo-Domenech J, Vidal SJ, Rodriguez-Bravo V, Castillo-Martin M, Quinn SA, Rodriguez-Barrueco R (2012). Suppression of acquired docetaxel resistance in prostate cancer through depletion of notch- and hedgehog-dependent tumor-initiating cells. Cancer Cell.

[26] Collins AT, Berry PA, Hyde C, Stower MJ, Maitland NJ (2005). Prospective identification of tumorigenic prostate cancer stem cells. Cancer Res.

[27] Luo J, Ok Lee S, Liang L, Huang CK, Li L, Wen S (2014). Infiltrating bone marrow mesenchymal stem cells increase prostate cancer stem cell population and metastatic ability via secreting cytokines to suppress androgen receptor signaling. Oncogene.

[28] Hernandez-Gea V, Toffanin S, Friedman SL, Llovet JM (2013). Role of the microenvironment in the pathogenesis and treatment of hepatocellular carcinoma. Gastroenterology.

[29] Paino F, La Noce M, Di Nucci D, Nicoletti GF, Salzillo R, De Rosa A (2017). Human adipose stem cell differentiation is highly affected by cancer cells both in vitro and in vivo: implication for autologous fat grafting. Cell Death Dis.

[30] Whiteside TL (2008). The tumor microenvironment and its role in promoting tumor growth. Oncogene.

[31] Thalmann GN, Rhee H, Sikes RA, Pathak S, Multani A, Zhau HE (2010). Human prostate fibroblasts induce growth and confer castration resistance and metastatic potential in LNCaP cells. Eur Urol.

[32] Yu Y, Zhang Q, Meng Q, Zong C, Liang L, Yang X (2016). Mesenchymal stem cells overexpressing Sirt1 inhibit prostate cancer growth by recruiting natural killer cells and macrophages. Oncotarget.

[33] Zhang T, Lee YW, Rui YF, Cheng TY, Jiang XH, Li G (2013). Bone marrow-derived mesenchymal stem cells promote growth and angiogenesis of breast and prostate tumors. Stem Cell Res Ther.

[34] Dogan A, Demirci S, Apdik H, Apdik EA, Sahin F (2017). Dental pulp stem cells (DPSCs) increase prostate cancer cell proliferation and migration under in vitro conditions. Tissue Cell.

[35] Liu S, Ginestier C, Ou SJ, Clouthier SG, Patel SH, Monville F (2011). Breast cancer stem cells are regulated by mesenchymal stem cells through cytokine networks. Cancer Res.

[36] Bharti R, Dey G, Mandal M (2016). Cancer development, chemoresistance, epithelial to mesenchymal transition and stem cells: a snapshot of IL-6 mediated involvement. Cancer Lett.

[37] Clarke MF, Dick JE, Dirks PB, Eaves CJ, Jamieson CH, Jones DL (2006). Cancer stem cells--perspectives on current status and future directions: AACR workshop on cancer stem cells. Cancer Res.

[38] Li X, Lewis MT, Huang J, Gutierrez C, Osborne CK, Wu MF (2008). Intrinsic resistance of tumorigenic breast cancer cells to chemotherapy. J Natl Cancer Inst.

[39] Folkins C, Man S, Xu P, Shaked Y, Hicklin DJ, Kerbel RS (2007). Anticancer therapies combining antiangiogenic and tumor cell cytotoxic effects reduce the tumor stem-like cell fraction in glioma xenograft tumors. Cancer Res.

