# Supplementary figures and images for: Mesenchymal stem cells recruited by castration-induced inflammation activation accelerate prostate cancer hormone resistance via chemokine ligand 5 secretion
Source: Stem Cell Res Ther. 2018 Sep 26;9:242. doi: 10.1186/s13287-018-0989-8 (PMC6158918; doi:10.1186/s13287-018-0989-8)

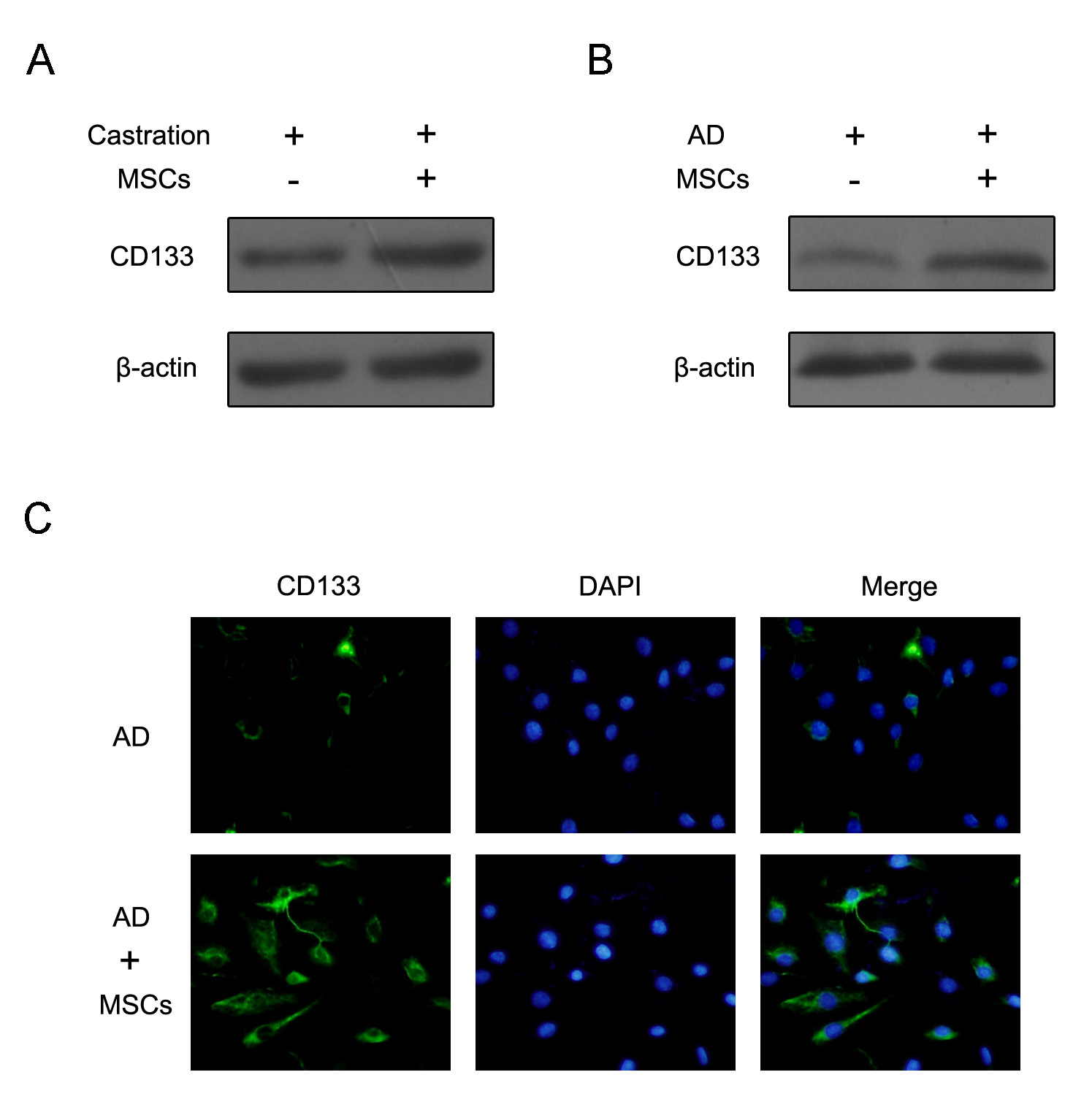

Supplement: Supplementary file 1 — Figure S1. MSCs increase stem marker expression of PCa. A. Western blot analysis of stem marker (CD133) expression in PCa tumor tissues. B. Western blot analysis of stem marker (CD133) expression in LNCaP cells. C. LNCaP cells stained with primary antibodies anti-CD133 (Abcam). Sections counterstained with DAPI (Beyotime) for nuclei staining. CD133 expression determined by fluorescence microscope. Typical photographs presented (original magnification: ×200) (JPG 205 kb) [file 13287_2018_989_MOESM1_ESM.jpg]
